# Green synthesis, characterization, molecular simulation, and in vitro biomedical application of magnesium oxide nanoparticles

**DOI:** 10.1371/journal.pone.0332367

**Published:** 2025-09-17

**Authors:** Samy Selim, Mohamed K. Y. Soliman, Mohammed S. Almuhayawi, Mohammed H. Alruhaili, Hattan S. Gattan, Amna A. Saddiq, Nashwa Hagagy, Ashwag Jaman Alzahrani, Soad K. Al Jaouni, Salem S. Salem

**Affiliations:** 1 Department of Clinical Laboratory Sciences, College of Applied Medical Sciences, Jouf University, Sakaka, Saudi Arabia; 2 Botany and Microbiology Department, Faculty of Science, Al-Azhar University, Nasr City, Cairo, Egypt; 3 Department of Clinical Microbiology and Immunology, Faculty of Medicine, King Abdulaziz University, Jeddah, Saudi Arabia; 4 Special Infectious Agents Unit, King Fahad Medical Research Center, King Abdulaziz University, Jeddah, Saudi Arabia; 5 Department of Medical Laboratory Sciences, Faculty of Applied Medical Sciences, King Abdulaziz University, Jeddah, Saudi Arabia; 6 Department of Biological Sciences, College of Science, University of Jeddah, Jeddah, Saudi Arabia; 7 Department of Biology, College of Science & Arts at Khulis, University of Jeddah, Jeddah, Saudi Arabia; 8 Botany and Microbiology Department, Faculty of Science, Suez Canal University, Ismailia, Egypt; 9 Department of Hematology/Oncology, Scientific Chair of Prophetic Medicine Application, Faculty of Medicine, King Abdulaziz University and Hospital, Jeddah, Saudi Arabia; K Ramakrishnan College of Technology, INDIA

## Abstract

Microbial infections represent a major hazard to global public health, resulting in extensive morbidity and mortality across the globe. As a result, in the past 10 years, nanoparticles have drawn a lot of interest in their potential to manage microbial diseases. One of the few studies that has used a green and environmentally acceptable approach of producing magnesium oxide nanoparticles (MgONPs) was employed via using an extract from watermelon peels. UV–visible, FTIR, XRD, and TEM were used to comprehensively characterize the biosynthesized MgONPs. The synthetic MgONPs have a polycrystalline form with a median particle size of 6–17 nm, according on the characterization of the material. According to the antimicrobial results, MgONPs showed notable antimicrobial properties toward B*. subtitles, S. aureus, E. coli, P. aeruginosa*, and *C. albicans*, with an inhibition zone measuring 18.2 ± 0.36, 23.7 ± 0.4, 15.4 ± 0.25, 17.6 ± 0.56, and 16.3 ± 0.32 mm respectively. While the minimum inhibitory concentrations (MICs) varied from 50 to 200 µg/mL. MgONPs have successfully demonstrated antibiofilm potential versus MRSA. A molecular docking simulation was carried out to obtain a better understanding of the potential mechanism of MgO-NPs against the *S. aureus* strain. The results imply that the activity may be attributed to the dihydrofolate reductase (DHFR) with a varying degree, and the predominant interaction observed is the hydrophobic interaction with the residues’ amino acids in the active site of the pocket in *S. aureus*. Furthermore, the DPPH technique revealed that MgONPs had considerable antioxidant activity, with an IC_50_ of 223 µg/mL. Additionally, at a dosage of 62.5 µg/mL, MgONPs exhibit possible antiviral efficacy against HAV and HSV1, with proportions of 84.7 and 49.7%, respectively. Finally, the watermelon peel extract biosynthesized MgONPs exhibit antimicrobial, antibiofilm, antioxidant, and antiviral properties that show promise to be utilized in the biomedical field.

## Introduction

Given that nanotechnology possesses so many uses in several scientific and technological domains, it has become a prominent subject of study recently [1,2]. It covers the formation of various types of nanoparticles (NPs) and how to use them in a range of industries, including biology, sensing, and catalysis [3]. Considering their remarkably high surface area compared to volume ratio, tiny particle size, changeable form, and high mechanical, optical, magnetic, and electrical qualities, among other attributes [4]. Nevertheless, the unique characteristics of a given NP are mostly determined by their synthesis method [5,6]. NPs may have produced in a variety of physical and chemical ways [7]. They need several hazardous chemical additions, high temperatures, vacuum conditions, and extremely complex instrumentation. However, the employment of many hazardous and reactive compounds in innovative chemical creation processes has also increased potential biological hazards to the ecosystem and the general populace. Significant effort has been devoted to an alternate and workable green synthesis method to produce numerous NPs to address these issues [8]. This preference arises because, compared to conventional chemical and physical techniques which often require hazardous substances and large amounts of energy, green synthesis is simpler, safer, more reliable, economical, and environmentally friendly [9–11]. The green or biosynthesis of NPs can be achieved using various plant materials, including roots, flowers, leaves, stems, bark, etc. [12,13]. The development of metal-based nanoparticles marks a significant advancement in nanotechnology, enabling the production of superior materials. Recently, researchers have successfully synthesized iron, silver, selenium, copper oxide, and gold nanoparticles using fruit and vegetable peels as well as their bagasse, which exhibit potent antibacterial and water-remediation properties [14,15]. The sustainable production of these nanomaterials depends on several factors, including the natural proportion of the metallic precursor, temperature, reaction duration, pH, and other reaction conditions [16]. Variations in these factors result in NPs with different sizes and morphologies, which greatly affect their physico-chemical as well as biological properties [17,18]. Metallic oxide nanoparticles (MNPs) are considered a significant group of widely used nanomaterials due to their unique chemical and physical-characteristics, as well as their many applications in biologica-medicine, food packaging, tissue engineering, catalysis, and environmental sciences [19,20]. Magnesium oxide nanoparticles (MgO-NPs), a type of metal oxide nanoparticle, have recently attracted considerable owing to their remarkable biomedical applications, biocompatibility, and exceptional stability under harsh-conditions [21,22].

MgO-NPs may readily engage with various biological-systems due to their beneficial physico-chemical properties, which include degree of ionicity, large surface-area, unique crystal structure, and oxygen deficiencies [23–25].Additionally, MgO-NPs have been explored as potential antifungal [26], antibacterial [27], antioxidant [28], anticancer [22] and antidiabetic agents [28]. Given the increasing prevalence of MgO-NPs in biomedicine, it is imperative to develop novel synthetic methods for their production. A thorough literature review reveals that the potential use of agricultural waste in the synthesis of MgO-NPs has not been extensively explored [29–31]. According to some studies, such waste could be converted into sustainable nanomaterials. The ideal biomass for cost-effective and environmentally friendly MgO-NPs synthesis would be a material with no competing beneficial uses. Watermelon peels, an abundant agricultural waste product, could serve as a viable source for this purpose.

**Fig 1:**
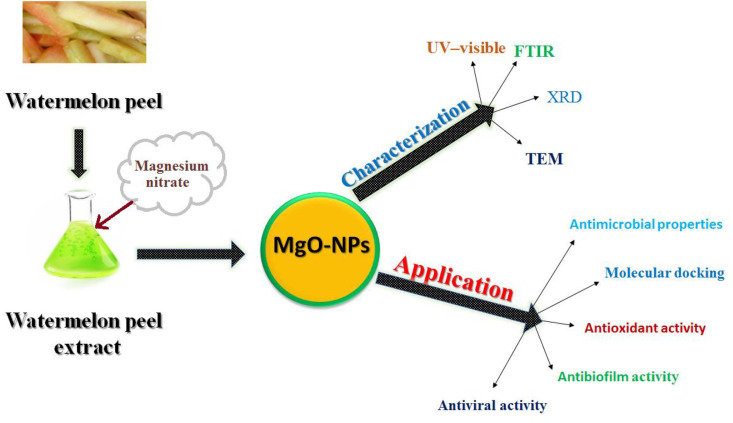
A schematic-diagram illustrating the production, characterisation, and applications of MgO-NPs synthesized by WP extract.

This study aims to utilize watermelon peel extract (WPE) for synthesis of magnesium oxide nanoparticles. The synthesized MgO-NPs were characterized using TEM, XRD, UV-Vis spectroscopy, and FT-IR. Furthermore, their antibacterial, antibiofilm, antioxidant, and antiviral properties were evaluated (Fig 1).

## Materials and methods

### Materials

Magnesium nitrate hexahydrate (Mg(NO₃)₂·6H₂O, ≥ 99.0%, Sigma-Aldrich, Cairo, Egypt. Sodium hydroxide (NaOH, ≥ 98%, pellets, Sigma-Aldrich, Cairo, Egypt). All media used for biological activities (antibacterial and antibiofilm) from Oxoid Dist., Cairo, Egypt. DPPH (2,2-diphenyl-1-picrylhydrazyl, ≥ 95%, Sigma D9132). In addition, the reagents or biological medium don’t need to be purified before use [32].

### Watermelon peels extract preparation

The watermelon was purchased from Carrefour market (30.174352611096854, 31.476022880013325) at Obour City, Qalyubia Governorate, Egypt. The watermelon peel (WP) was cleansed with thoroughly distilled water many times, and the same technique was used to establish the extract as in a previous study with a few little adjustments [33]. Next, the peel (the white portion alone) was sliced into little pieces (about 1 cm) and placed in 200.0 g with 1 L of d. water. Following utilizing a magnetic stirrer to continuously agitate the mixture for 10 min, employing Whatman no. 1 filter paper, WPE had been cooled, collected into an uncontaminated bottle, then kept at 4°C as long as required.

### Biosynthesis of MgO-NPs

Following the last study with a few minor adjustments, the Sharma et al. [34] technique was ensued in order to generate MgO-NPs. In summary, 50 mL of freshly prepared magnesium nitrate (1.0 mM) solution was combined with Fifty mL of watery WP extract. The mixture was then mixed with ten milliliters of drops of added sodium hydroxide (1.0 M) at 80°C. For four h., the solution was continuously stirred at 500 rpm using a magnetic-stirrer. The colour transformation from pale yellow to brownish served as an indication to start generating nanoparticles. Next, the produced MgO-NPs were calcined for 3-h at 200°C to produce the dry MgO-NPs and the resulting powder was maintained in the dark for further-investigation.

### Characterization of generated MgO-NPs

A variety of modern analytical techniques were employed to characterize the biosynthesized MgO-NPs. Initially, a shift in colour was used to visually observe the creation of MgO-NPs. Employing a spectrophotometer (JENWAY 6305 Spectrophotometer), UV-Vis spectroscopy, the colour change resulting from the synthesis of NPs exhibiting a wavelength of 200–700 nm [35]. The structural features of the generated MgO-NPs were investigated utilizing a TEM (JEOL- 1010 -Japan). After adding a drop of the MgO-NPs onto the C-coated Cu- grid, it was let dry out until being set on a holder. Furthermore, it was allowed to image the specimen that was gathered. With the use of FT-IR spectroscopy (Agilent system Cary 660 FT-IR model), the goals of the functional groups in charge of the reduction, capping, and stability of MgO-NPs were evaluated. The 400–4000 cm^−1^ region of FT-IR spectra was used to analyze the materials [36]. Furthermore, the crystalline form of biosynthesized MgO-NPs was examined using the X-ray diffractometer X′ Pert Pro (Philips, Eindhoven, the Netherlands) and designs created by XRD. The range of values for 2θ was 4^o^ to 80^o^ on average. Cu Ka radiation that had been Ni-filtered was the source of the X-rays. A 40 kV voltage and a 30 mA current were present, respectively. WP extract’s median size of MgO-NPs was determined by applying the Debye-Scherrer equation [37].

### Antimicrobial properties of MgO NPs

Utilizing the agar well diffusion technique, the antibacterial activity of the MgO-NPs was assessed toward *Pseudomonas aeruginosa* ATCC 9022, *Escherichia coli* ATCC 8739, *Bacillus subtilis* ATCC 6633, *Staphylococcus aureus* ATCC 6538, and *Candida albicans* ATCC 10231. Muller Hinton agar (MHA) and tryptic soy agar (TSA) serve as the mediums employed for observing the growth of the microbial strains (Oxoid, USA). After a day, the suspension of microbes was added to autoclaved medium that had an average density of half of the McFarland standard. After that, they were divided equally across the Petri dishes. With the use of a sterile cork-borer, an 8 mm hole was made. Next, 100 μl of the MgO-NPs (1000 μg/ml) had been added to the well, and it was incubated at 37°C for 24 h. In the same way, WP extract and magnesium nitrate were used as negative control tests. Amoxicillin (1000 μg/ml) functioned as the positive- control for bacterial tests, while Fluconazole (1000 μg/ml) was used as the positive-control for fungal test. Using a digital caliper, the region of suppression was determined after incubation [38]. The diameters of the zones were expressed in mm. The outcome was recorded in compliance with CLSI criteria [39].

The MIC of MgO-NPs against pathogenic microorganisms was determined using the broth microdilution method [36]. Different amounts of MgO-NPs have been generated, ranging from 800 μg/mL to 12.50 μg/mL. Sterilized MTP wells were filled with MgO-NPs to be evaluated at various concentrations after 100.0 μL of double-strength Mueller hinton (MH) broth was supplied. A microbial-suspension of cells (20 μL) matching the 0.5 McFarland standard has been added, excluding the negative and positive control wells. For twenty-four hours, the plates are incubated at 37°C. The MIC was determined in accordance with the Clinical and Laboratory-Standards Institute (CLSI) guidelines using a microplate-reader (STAT-FAX, USA) and the least quantity of samples that suppressed the test pathogens in a way that was analogous to negative as well as positive controls [40].

### Anti-Biofilm activity of biosynthesized MgO-NPs

The microtiter plate (MTP) method was employed to assess the anti-biofilm activity of MgO-NPs against *S. aureus* MRSA, a clinical strain renowned for its strong biofilm-forming capacity. A few changes were made to the biofilm- experiment from the last study. In summary, MgO-NPs were introduced in varying quantities to MTP, including TSB Media that were enriched with 1% glucose. The experimental microorganism was set up on MTP over 48 h at 37.0°C after being diluted 1:100 in TSB. Following the incubation time, the development density (OD at 620nm) had been determined before removing the planktonic cells onto the dishes. Subsequently, wells were gently washed 3-times with phosphate-buffered saline (PBS) to preserve intact biofilms. Biofilms were fixed with 200 μL of 95% methanol for 10 min, followed by staining with 0.3% crystal violet (200 μL/well) for 20 min at room-temperature. Excess stains were removed by washing with distilled- water, and biofilm-associated dye was solubilized with 30.0% acetic Acid for quantification. Employing a microplate- reader (STAT-FAX-USA) set to O.D. at 540 nm, the absorbance was obtained. By comparing to control wells, the results were confirmed [41].

### Molecular docking

**Fig 2:**
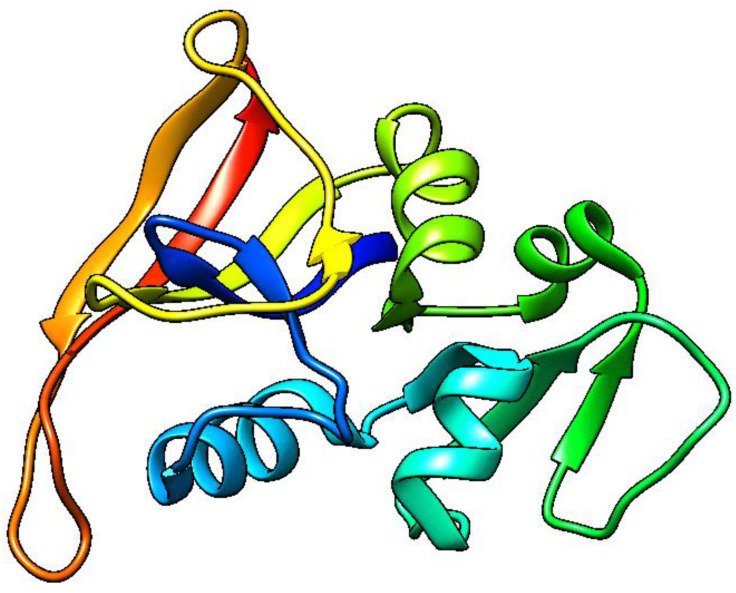
3D crystallography of the DHFR enzyme obtained from protein data bank.

The Molecular Operating -Environment (MOE) version MOE- 2019.0102 was utilized to carry out the molecular docking simulation procedure for MgO-NPs. Target receptor dihydrofolate reductase’s 3D structure (PDB: 2w9h) (Fig 2) was obtained from the protein -data bank using the following link: https://www.rcsb.org/structure/2veg (last accessed 4/10/2024). By applying the previously described method [42], just one chain (chain- A) was needed to build the receptor’s active site. The construction of dihydrofolate reductase (PDB: 2w9h) involved the generation of an active site through the selection of (chain A) and subsequent generation in accordance with standard protocol. The docking process was completed using triangle- matcher placement, London-dG, and GBVI/WSAdG- as Rescoring-1 and 2, respectively. In addition, Forcefield was used for post-placement refinement. The docking attitude exhibited the most binding energy, with a negative value. In addition, the MgO-NPs structure was created using the identical procedure as previously reported [42].

### Antioxidant activity of biosynthesized MgO-NPs

To test MgO-NPs’ antioxidant properties, the DPPH technique was applied [43]. The ability of bio-synthesized MgO-NPs to scavenge DPPH radicals was tested at various doses (1000 to 7.81 μg/mL). The antioxidant capability of ascorbic acid (AA) was assessed using the methodology of Almuhayawi et al., [44], and the subsequent equation has been employed to compute the DPPH- scavenging activity (%) for different quantities of MgO-NPs.

DPPH− scavenging activity (%)= A1)− A2)/ A1×100

A1): Absorbance of control, A2): Absorbance of test

### Antiviral activity of biosynthesized MgO-NPs

To determine (MNTC of the synthesized MgO NPs, the particles were added to a 96-well plate containing standard Vero cells (ATCC CCL-81, Science Way Co., Cairo, Egypt) as host cells. The MTT assay was then used to calculate the MNTC. A confluent monolayer of Vero cells was prepared by seeding 10⁴ cells/well in 100.0 µL growth medium, followed by incubation in a 5% CO₂ incubator at 37°C for 24 h. After incubation, the growth medium was removed, and the cells were treated with MgO-NPs at concentrations ranging from 1000 to 31.25 µg/mL. Untreated cells served as the negative control in the triplicate tests of MgO-NPs dissolved in DMSO at each dose. In a 5% CO₂ condition, treated plates were left to stand for 48 h at 37.0°C. After incubation, each well received 20 µL of MTT- solution, which was agitated for 5 min at 150.0 rpm and then incubated for 5 h at 37°C with CO_2_. Following supernatant removal, 200 µL of DMSO was added to each well to dissolve the formed formazan crystals. The absorbance was subsequently recorded at 570 nm using a microplate ELISA reader. The MNTC was determined by plotting the relationship between MgO-NPs concentrations and cell viability percentages.

The antiviral efficacy of MgO-NPs against the herpes simplex-virus (HSV-1) and hepatitis A- virus (HAV) was evaluated. A confluent monolayer of Vero cells was prepared, and a viral-suspension was incubated with MgO NPs (1:1 (v/v) for 1 h at 35.0 ± 2°C. Then, 100.0 µL of the virus-NP mixture was added to each well. Untreated Vero cells (non-infected) served as the control. The plate was shaken at 150.0 rpm for 5 min and incubated overnight at 37.0°C with 5% CO₂. Cell viability was assessed by measuring formazan crystal absorbance after adding MTT solution. In the same way, Acyclovir and Amentadine served as the positive controls for HSV-1 and HAV tests, respectively. By contrasting the optical density (OD) values of live infected cells with those of uninfected controls, the effectiveness of the antiviral treatment was evaluated [45].

### Statistical analysis

Three replications’ means and the standard deviation (SD) were calculated for each of the outcomes, and the data thereafter underwent an analysis of difference utilizing one-Way and two-Way ANOvA at Graph Pad prism-version 8.0.2.(p-value < 0.05 (*), p-value < 0.01 (**), p-value < 0.001(***), p-value < 0.0001(****))

## Results and discussion

### Biosynthesis of MgO-NPs using WP extract

Fruit peels, typically discarded as waste, are an excellent-source of phytochemicals that play a crucial role in nanomaterial synthesis. When plant organ extracts were used for nanoparticle production, varied results were observed. In our study, MgO-NPs formed after 4 hours of incubation, with visual inspection revealing a steadily increasing synthesis rate. Following nanoparticle formation, the solution colour changed from clear to yellowish-brown, indicating successful synthesis. The watermelon peel (WP) extract served as both a reducing and capping agent, enhancing the colloidal stability of the NPs. Even at low concentrations, the biomolecules present in the extract not only reduced the number of metal ions required for nanoparticle formation but also prevented aggregation of the synthesized NPs [46]. The optical properties of the colloidal nanoparticle solution varied depending on the particle size and shape, primarily due to surface-plasmon-resonance (SPR). Key phytochemicals in fruit peels—such as aldehydes, flavones, amides, terpenoids, and sugars—facilitate bioreduction and NP stabilization [47].

### Characterization of biosynthesized MgO-NPs

**Fig 3:**
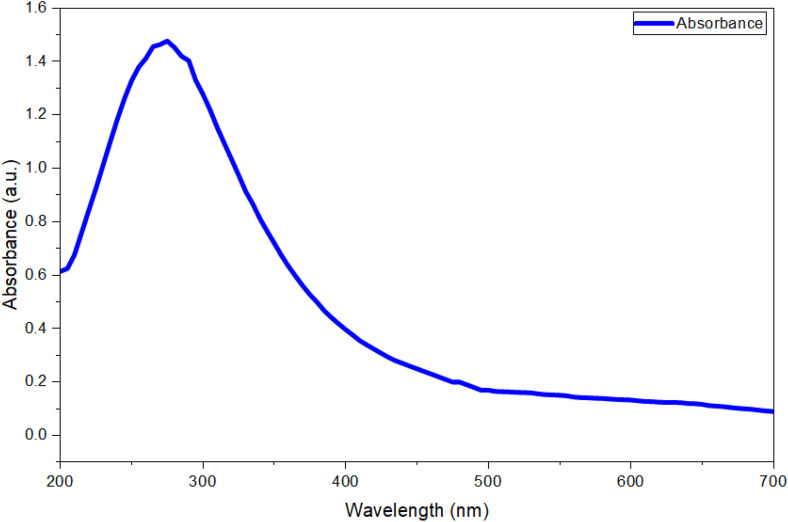
UV-vis spectrum of biosynthesis MgONPs.

Research has confirmed that the phytochemical components present in watermelon peel (WP) extract facilitate the synthesis of MgO-NPs. When the WP extract reduced magnesium ions in solution, MgO-NPs were formed. The first observable evidence of successful biosynthesis occurred when the mixture of aqueous WP extract and magnesium nitrate salt changed colour from light yellow to yellowish-brown. This colour transformation resulted from surface plasmon resonance (SPR), a characteristic optical property of MgO-NPs, with absorption maxima (λₘₐₓ) in the 260–300 nm visible range [48]. The generated nanoparticles’ dimensions, morphologies, nature, excellent dispersion, particle-to-particle separation, and external medium all had a substantial impact on the SPR absorbance [49]. According to Jeevanandam et al., the generated MgO-NPs appear to be lower in diameter at SPR frequencies not more than 300 nm; still, the degree of anisotropy increases for SPR greater than 300 nm [50]. The present investigation evaluated the optical- properties of MgO-NPs developed via WP extract mediated at wavelengths spanning from 200 to 700 nm. MgO-NPs UV-Vis spectra showed the existence of nanoscale particles, with a resonance band at 275 nm (Fig 3). The finding is displayed as associated with other researchers [51,52]. The most noticeable bands of MgO-NPs derived from *Tecoma stans* (L.) and *Rosmarinus ofcinalis* L were found at 282, and 250 nm, respectively, according to these investigations [51,52]. Furthermore, MgO-NPs made chemically had a measurable absorbance band at 290 nm [53].

**Fig 4:**
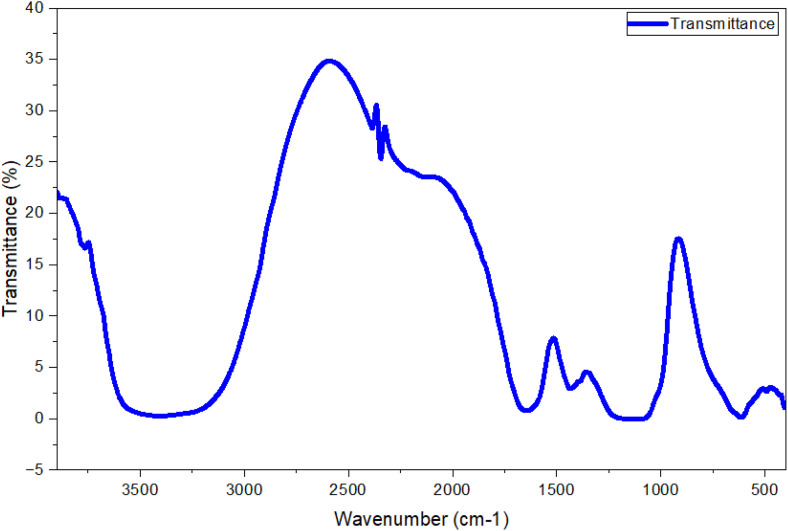
FT-IR spectra of biosynthesized MgONPs.

Fourier-transform infrared (FTR)-spectroscopy (4000–400 cm ⁻ ¹) revealed that the WP extract performed two responsibilities in the process of MgO-NPs, modelling as both a capping agent and reducing agent for the participating functional groupings. The FTIR spectrum of the biosynthesized MgO-NPs exhibited characteristic absorption bands at 3406 cm ⁻ ¹ O–H stretching, 2354 cm^-1^ and 2345 cm^-1^ C–H vibrations, 1641 cm^-1^ C = C, *cis* configuration, 1423 cm^-1^ and 1384 cm^-1^ C–O bonds, 1174 cm^-1^ and 1100 cm^-1^ flavonoids/tannins 616 cm^-1^, 487 cm^-1^, and 405 cm^-1^ Mg–O vibrations (Fig 4). These bands confirmed the presence of bioactive metabolites phenolics, carbohydrates, flavonoids, and tannins adsorbed on the NP surface [54]. The stability and formation of MgO-NPs depended on these functional molecules. Additionally, two distinct peaks at 635 cm ¹ and 495 cm ¹ were attributed to Mg–O vibrational modes, confirming MgO-NPs formation [55]. This finding aligns with previous reports identifying the 437–677 cm^-1^ range as characteristic of MgO-NPs synthesis [56].

**Fig 5:**
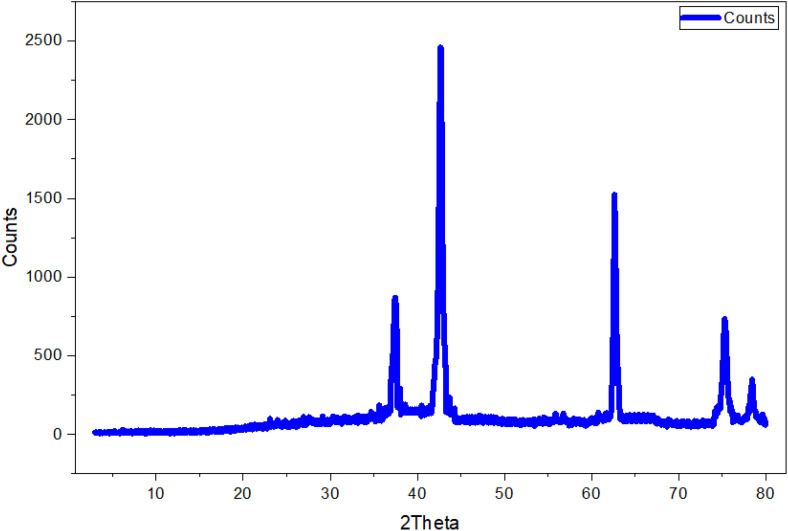
XRD analysis of biosynthesized MgONPs.

The X-ray Diffraction (XRD) analysis confirmed both the crystalline-structure and phase purity of the biosynthesized MgO-NPs. The diffractogram (Fig 5) showed distinct diffraction peaks, verifying the crystalline-nature of MgO-NPs. The diffraction pattern revealed well-defined reflection of planes and a cubic crystal structure. The detected peaks confirmed the high-purity of the synthesized MgO-NPs, as they corresponded exclusively to the cubic phase of MgO without any secondary phase impurities. Five distinctive peaks were seen at 2θ values of 37.14°, 42.71°, 62.66°, 77.29°, and 78.46°, which were linked to the (111), (200), (220, (311, and (222) reflection planes, respectively. Additional peaks at 36.5° and 75.8° (corresponding to (111) and (311) planes) indicated the existence of trace levels of Mg(OH)₂. The broadening of peaks at (200), (220), and (222) suggested the formation of smallnanosized cubic MgO-NPs [57]. This XRD pattern was consistent with previous reports of green-synthesized MgO-NPs using various bioactive compounds [58,59].

**Fig 6:**
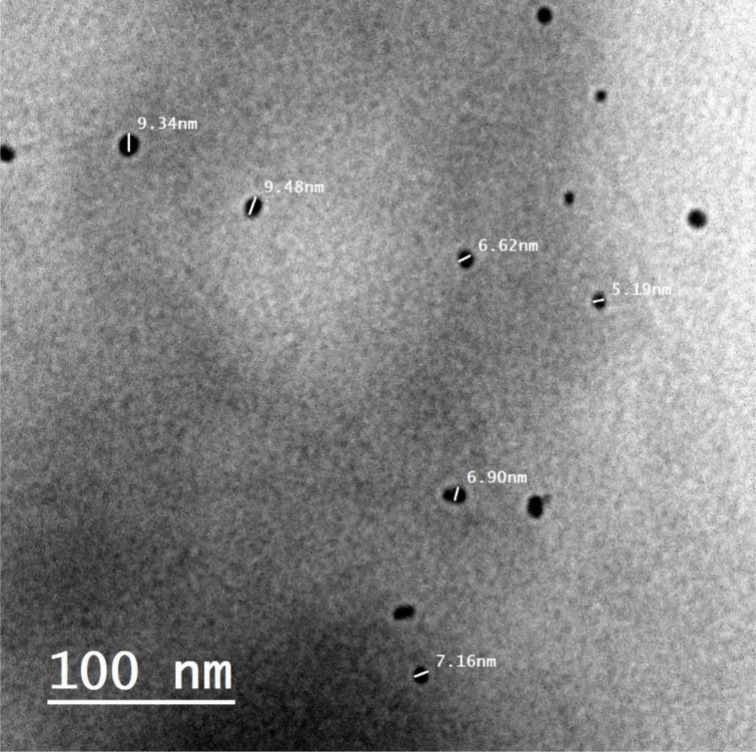
TEM image of the biosynthesized MgONPs.

The dimensions and form of NPs are often connected to their activity, since their performance increases progressively as the size decreases. The produced MgO-NPs’ size formation and surface structural characteristics were examined using HR-TEM. In line with other research, the HR-TEM micrograph of MgO-NPs (Fig 6) clearly showed the spherical form of biosynthesized MgO-NPs [60]. Using a TEM at 100 nm, the generated MgO-NPs’ nanometer size ranged from 6 to 17 nm. The properties of metabolites, including proteins and enzymes, in forming and altering the morphologies of specific-NPs, such as circular, cubic, and hexagonal, along with rods, are the main problems that require immediate attention and more research [61].

### Antimicrobial effectiveness of Biosynthesized MgO-NPs

**Fig 7:**
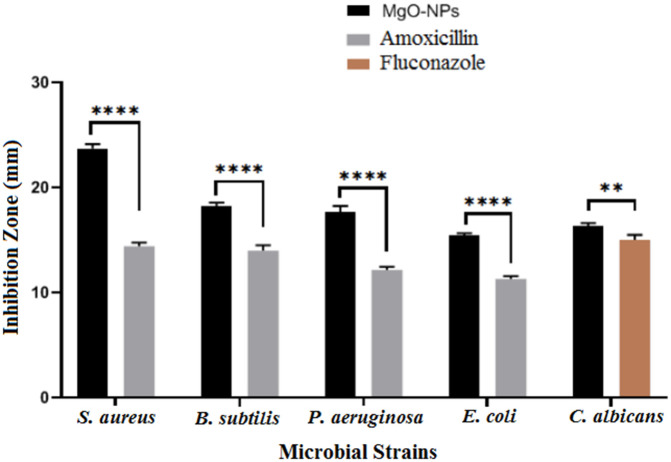
Antimicrobial properties of MgONPs versus different microbial strains.

Antimicrobial resistance (AMR) remains a major challenge in clinical infectious disease management. In accordance with the standards of the Clinical and Laboratory Standards-Institute (CLSI), the antibacterial activity of biosynthesised MgO-NPs was assessed using the agar diffusion technique. Inhibition zone diameters (mm) were measured to assess antimicrobial efficacy. As shown in Fig 7, the MgO-NPs exhibited significant antimicrobial activity, evident by clear inhibition zones. At 1000 μg/mL, the NPs demonstrated strong activity against *Bacillus subtilis* (18.2 ± 0.36 mm) and *Staphylococcus aureus* (23.7 ± 0.4 mm) (S1 Table). The lowest activity was observed against *Escherichia coli* (15.4 ± 0.25 mm) and *Candida albicans* (16.3 ± 0.32 mm). In contrast, *Pseudomonas aeruginosa* showed moderate susceptibility (17.6 ± 0.56 mm). These results may be attributed to the small size of MgO-NPs (32–39 nm), which has been previously reported to enhance antimicrobial effects against Gram-negative bacteria [60]. Notably, MgO-NPs synthesized using *Psidium guajava* leaves have demonstrated potent bactericidal properties [62]. Yoon et al. [63] and Das et al. [64] similarly reported that higher MgO-NPs concentrations increased antibacterial efficacy, particularly against *E. coli*, in a dose-dependent manner. The MIC values of MgO-NPs were determined as follows, 100 μg/mL for *B subtilis* and *P aeruginosa,* 200 μg/mL for *Ecoli* and *C albicans* and finally 50 μg/mL for *S aureus.* The antibacterial effect of MgO-NPs arises from the attraction between Mg^2+^ ions and the negatively charged bacterial cell surface, which causes damage to the cell wall, leads to protein malfunction, and results in cell death [65]. Adsorption of Mg^2+^ ions to membrane proteins, causing proton pump dysfunction and loss of membrane integrity [66]. Intracellular penetration of MgO-NPs, resulting in ATP depletion, reactive oxygen-species (ROS) generation, DNA/protein damage and membrane leakage [67]. Additionally, Sawai et al. [68] proposed that MgO-NPs adsorb water molecules, forming an alkaline surface layer (high pH) that damages bacterial membranes and induces cell death.

### Anti-biofilm of Biosynthesized MgO-NPs

**Fig 8:**
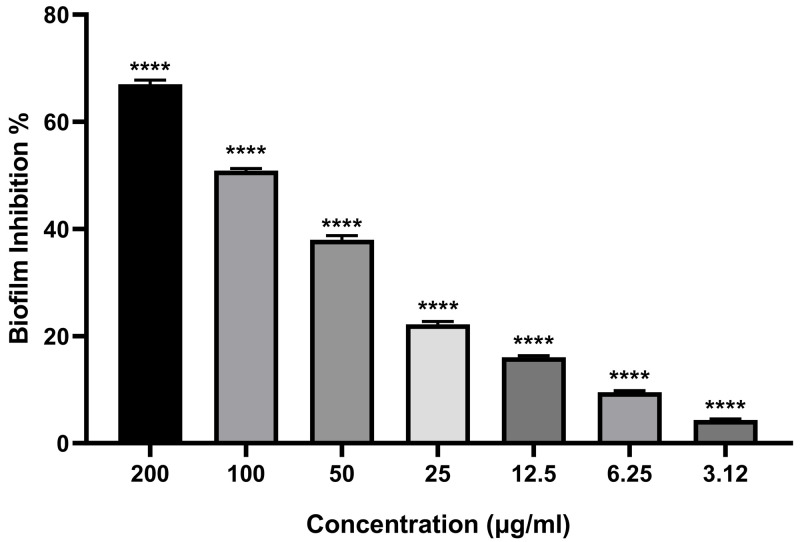
Antibiofilm activity of biosynthesized MgONPs toward MRSA at varying doses.

**Fig 9:**
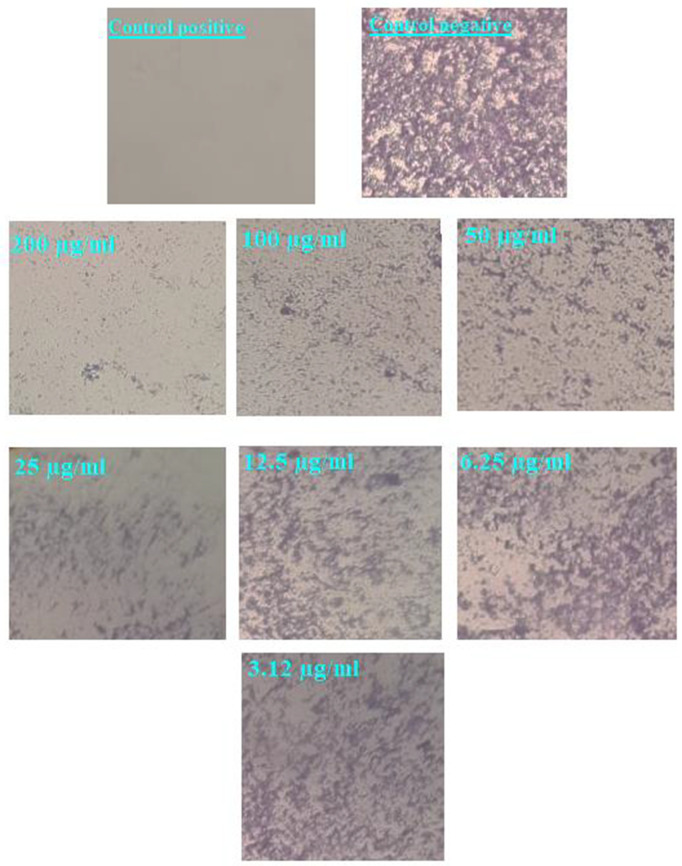
Light-inverted microscopy pictures of *S. aureus* biofilms cultivated in different MgONPs doses.

Biofilms are microbial communities that form surface-adherent structures, significantly enhancing microbial resistance to antimicrobial treatments [69]. These complex matrices consist of proteins, fibrin, and polysaccharides [70]. Notably, antibiotics effective against planktonic bacteria are often 10−1000 times less potent against bacterial biofilms [71]. In this study, MgO-NPs demonstrated dose-dependent antibiofilm activity against MRSA (S2 Table). The inhibition rates were 67.1% at 200 µg/mL, 50.9% at 100 µg/mL, 38.1% at 50 µg/mL, 22.2% at 25 µg/mL and 4.3% at 3.12 µg/mL (Fig 8). Light microscopy confirmed biofilm disruption, showing dissolution of microcolonies across all tested concentrations (200–3.12 µg/mL) (Fig 9). These findings align with Khan et al.‘s results using the crystal violet assay [72]. MgO-NPs were more effective at preventing biofilm formation than eradicating established biofilms [73]. Compared to ciprofloxacin, MgO-NPs showed superior antibiofilm activity against *P. aeruginosa*, *S pyogenes* and *S epidermidis* [74]. Biofilm inhibition concentrations were sub-MIC doses (0.5 × MIC) significantly reduced mature biofilms for *S. aureus* 500 µg/mL, *Klebsiella pneumoniae* 125.0 µg/mL and *E. coli*: 250 µg/mL [75]. Low concentrations (10 µg/mL) effectively prevented *S. aureus* biofilm formation [76]. Smaller MgO-NPs (8 nm) showed enhanced inhibition against *S aureus* and *Ecoli* biofilms [77].

### Docking simulation

**Fig 10:**
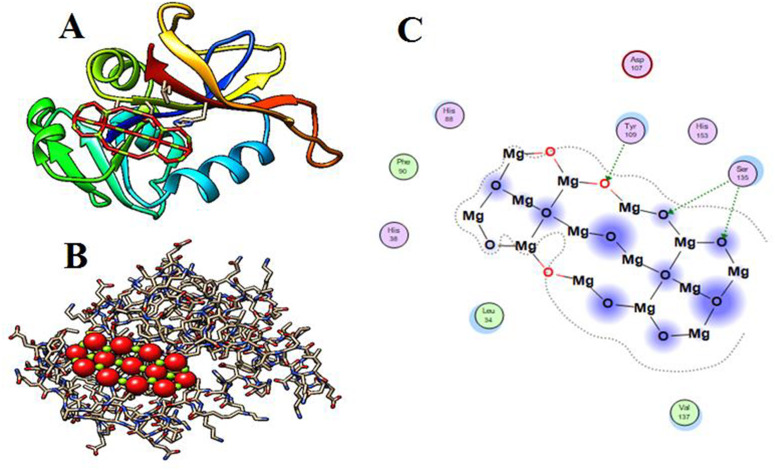
Structural interaction of MgO-NPs within the active site of dihydrofolate reductase (DHFR) (PDB ID: 2W9H). (A & B) 3D representations showing the binding mode of MgO-NPs within the DHFR active site, highlighting interactions with surrounding amino acid residues. (C) 2D interaction diagram illustrating the binding of MgO-NPs within the active pocket and their interactions with key amino acids.

Molecular docking- simulation is a powerful computational tool for predicting the 3D structure of protein-ligand-complexes and analyzing their interaction mechanisms [78]. This technique also provides insights into the potential modes of action and biological responses of nanomaterials. In this study, we performed docking simulations of MgO-NPs with the active site of DHFR (PDB: 2W9H) from *Staphylococcus aureus* (Fig 1). Finding out how specific enzymes interacted with MgO-NPs was the aim of the study, which aimed to discover potential targets for antibacterial action. Initially, the DHFR (PDB: 2w9h) and the co-crystallized ligand’s root mean square-deviation (RMSD) values were determined. They were 0.7813 and 0.9317 Å, respectively. The dihydrofolate reductase (DHFR) (PDB: 2W9h) was shown to display hydrophobic contact inside the active site of the MgO-NPs with various amino acid residues, including Tyr109 and Ser135 (Fig 10), and binding energy S = −3.3015 kcal/mol. We can state that MgO-NPs demonstrate their antibacterial activity through the inhibition of dihydroorotase synthase. However, the binding energy suggests that MgO-NPs favour DHFR more, and their activity is indicated by hydrophobic-interactions in the pocket containing the active site, which is located less than 2.7 Å away. The effectiveness and binding affinity of the MgONPs with the *Staphylococcus aureus* DHFR enzyme were assessed, along with the postulated method of binding to bacterial cells [79]. The most important enzyme for the bacterial species’ survival is DHFR. It is necessary for cell development and proliferation and is extensively expressed in bacterial species. It is hypothesised that interactions between nanoparticles and this enzyme prevent DNA replication in this species [80]. Consequently, antibacterial medications in synthetic materials may exert their effects by forming favorable interactions with the amino acid residues of target enzymes.

### Antioxidant activity

**Fig 11:**
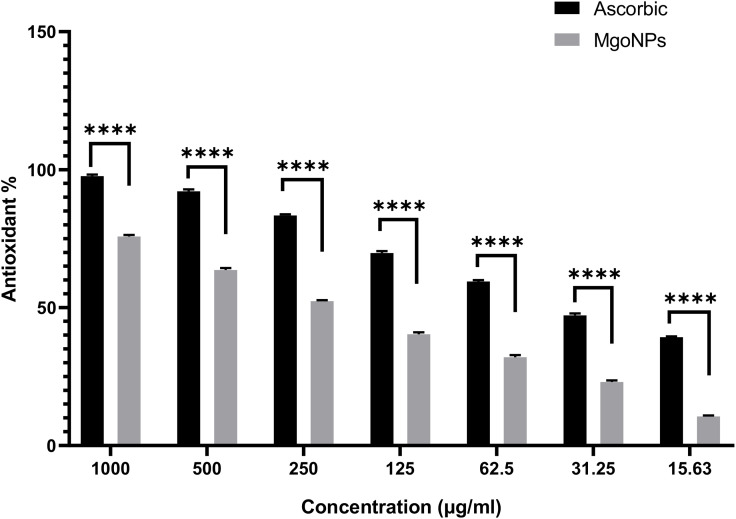
Antioxidant capability of biosynthesized MgONPs at varies concentrations.

Fig 11 presents the antioxidant potential of MgO-NPs evaluated across a range of concentrations.UV-Vis spectroscopic analysis revealed distinct absorbance peaks at 517 nm, corresponding to DPPH radical scavenging activity. The scavenging efficiency increased proportionally with NP concentration, showing values of 75.8, 63.6, 52.3, 40.3, 32.1, 23.2, and 10.5% from 1000–15.63 µg/mL, correspondingly (S3 Table), are the percentages of DPPH scavenging of MgO-NPs at the highest doses and the IC50 of MgONPs was recorded at 223 µg/mL. The antioxidant mechanism involves electron transfer from MgO-NPs to DPPH radicals, converting the solution from purple to yellow, which confirms the strong antioxidant capacity of the synthesized NPs [81]. The enhanced antioxidant-properties of MgOـNPs can be attributed by the existence of additional flavonoids as well as phenolics as capping on the outer layer of the magnesium oxide NPs derived from extract [82]. Using a DPPH method, the antioxidant activity of magnesium oxide nanoparticles generated by *Clitoria ternatea* has been assessed. It was discovered that at a dose of 150 mg/ml, the biologically produced MgO-NPs’ DPPH activity reach to 65% [83]. Using the DPPH test, Dobrucka et al. also investigated the antioxidant activity of produced MgO-NPs and discovered that they were antioxidants [84]. Additionally, free radicals such H_2_O_2_, O_2_^−^, and OH• as well as reactive-oxygen species (ROS) may be produced by NPs. Highly reactive substances called free radicals and ROS produce intense oxidative stress inside cells, which damages DNA, destroys proteins, and eventually results in cell death [85]. Most ROS are transformed into hydroxyl radicals [86]. Metal catalysts can decompose superoxide (O_2_^−^) and hydrogen-peroxide (H_2_O_2_), a process that generates hydroxy-radicals (•OH) [87]. This reactivity stems from the unpaired electrons in certain metals, which facilitate single electron transfer reactions. When hydroxyl radicals interact with DNA’s sugar backbone, they abstract hydrogen atoms from each carbon, forming carbon-centered sugar radicals. These reactive intermediates subsequently cause base-free sites, DNA strand breaks and modified sugar products [88]. Biologically synthesized nanoparticles exhibit superior antioxidant capacity compared to those produced through chemical or physical methods. This enhancement is attributed to plant-derived capping agents, which consist of alkaloids, polysaccharides, flavonoids and phenolic compounds. These phytochemicals synergistically enhance the nanoparticles’ antioxidant potential [89].

### Antiviral Assessment of Biosynthesized MgO-NPs

**Fig 12:**
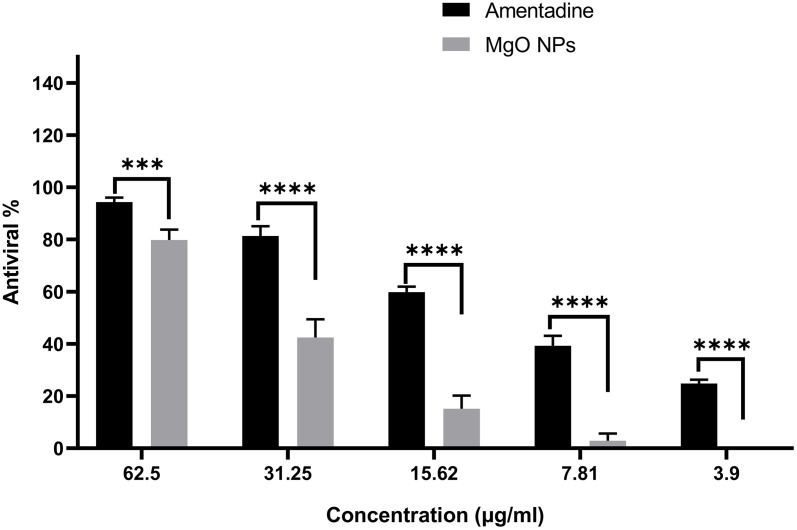
Antiviral efficacy of bioـsynthesized MgONPs against HAV.

**Fig 13:**
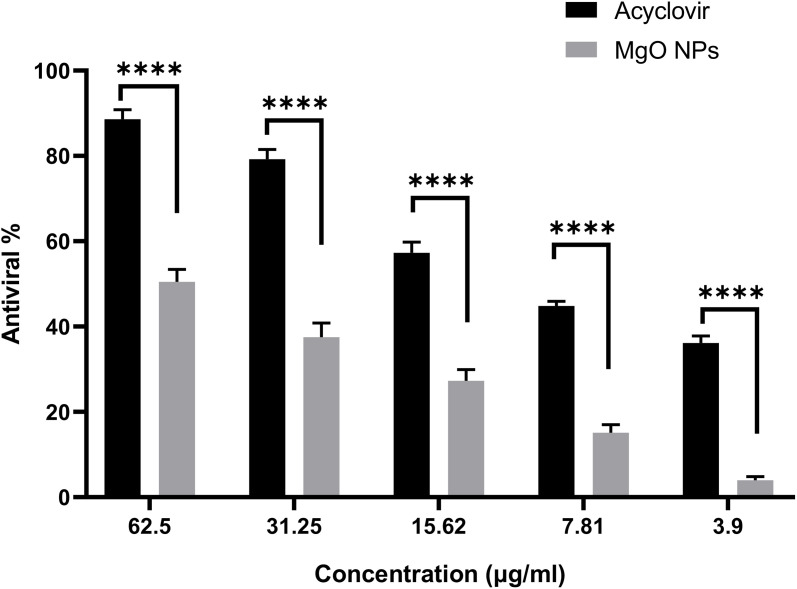
Antiviral efficacy of bioـsynthesized MgONPs against HSV1.

This study assessed the antiviral effectiveness of MgO-NPs biosynthesized using WPE against hepatitis A-virus (HAV) and herpes simplex-virus 1 (HSV-1) (S4 Table). The cytotoxic potential of MgO-NPs was first examined using normal Vero cell lines, revealing a maximum non-toxic concentration (MNTC) of 62.5 µg/mL. Using the MTT antiviral assay, we demonstrated that Vero cell viability improved when treated with green-synthesized MgO-NPs in virus-infected cultures, compared to virus-only controls. Data analysis showed these biogenic MgO-NPs exhibited 84.7% antiviral activity against HAV and 49.7% against HSV-1 (Figs 12 and 13). It seems that there are several different ways that nanoparticles may break down or deactivate viral particles [67]. For example, they can break the disulfide bonds that keep viruses structural, or they can release metal ions which combine with viral protective envelopes to produce ROS that harm viruses’ vital biological macromolecules like proteins and nucleic acids [90]. Furthermore, infected cells may create ROS in response to NP exposure. These ROS can damage viral proteins, DNA, and lipid membranes, which eventually prevents the virus from replicating and infecting new cells [91]. Certain authors have found that coating substances, such flavonoids, alkaloids, and terpenoids, that increase their antiviral properties can impact the antiviral effects of green-produced NPs [92,93].

## Conclusion

For the first time, WP extract was utilized in the eco-friendly synthesis of MgO-NPs production. The average size range of 6–17 nm. This green approach is environmentally safe, as it avoided hazardous chemicals and high-energy processes. The biosynthesized MgO-NPs demonstrated significant antibiofilm, antioxidant, and antibacterial action against tested bacteria as well as the unicellular fungal pathogen *C. albicans*. Additionally, at acceptable doses, MgO-NPs exhibit antiviral efficacy against HAV and HSV1. The enzyme hybrid MgO-NPs complex is stable, as demonstrated by the docking simulation of MgO-NPs, and the DHFR may be a potential mechanism of action. MgO-NPs represent a promising therapeutic candidate, pending further in vivo validation.

## Supporting information

S1 File Antimicrobial activity of MgO-NPs at 1000 µg/mL. (PDF)

S2 File Anti-biofilm assay of MgO-NPs. (PDF)

S3 File Antioxidant assay of MgONPs. (PDF)

S4 File Antiviral activity of MgONPs against HSV1 and HAV viruses. (PDF)
