# Supplementary material for: Green synthesis, characterization, molecular simulation, and in vitro biomedical application of magnesium oxide nanoparticles
Source: PLoS One. 2025 Sep 17;20(9):e0332367. doi: 10.1371/journal.pone.0332367 (PMC12443314; doi:10.1371/journal.pone.0332367)
Supplement: S1 File — (PDF) [file pone.0332367.s001.pdf]

S1: Antimicrobial activity of MgO-NPs at 1000 µg/mL.

Strains	MgO-NPs at 1000 µg/mL.				
	R1	R2	R3	Mean	SD
S. aureus	23.3	23.8	24.1	23.73333	0.404145
B. subtitles	18.2	18.57	17.85	18.20667	0.360046
P. auroginosa	18.3	17.55	17.2	17.68333	0.561991
E. coli	15.65	15.4	15.15	15.4	0.25
C. albicans	16.6	16.31	15.95	16.28667	0.325628
Amoxicillin (1000 µg/mL)					
S. aureus	14	14.5	14.7	14.4	0.36
B. subtitles	13.5	14	14.5	14	0.5
P. auroginosa	12	12.5	12	12.1666	0.2886
E. coli	11	11.5	11.4	11.3	0.2645
Fluconazole (1000 µg/mL)					
C. albicans	15	14.5	15.5	15	0.5
