# Supplementary material for: Green synthesis, characterization, molecular simulation, and in vitro biomedical application of magnesium oxide nanoparticles
Source: PLoS One. 2025 Sep 17;20(9):e0332367. doi: 10.1371/journal.pone.0332367 (PMC12443314; doi:10.1371/journal.pone.0332367)
Supplement: S2 File — (PDF) [file pone.0332367.s002.pdf]

S2: Anti-biofilm assay of MgO-NPs.

MgO-NPs	Inhibition %				
Conc (μg/mL)	R1	R2	R3	Mean	SD
200	67.9	66.5	66.7	67.03	0.757
100	51.3	50.8	50.6	50.9	0.361
50	38.9	37.5	37.8	38.07	0.737
25	22.78	22.1	21.7	22.19	0.546
12.5	16.32	16.1	15.8	16.07	0.261
6.25	9.86	9.45	9.21	9.507	0.329
3.12	4.56	4.3	4.11	4.323	0.226
