# Supplementary material for: Green synthesis, characterization, molecular simulation, and in vitro biomedical application of magnesium oxide nanoparticles
Source: PLoS One. 2025 Sep 17;20(9):e0332367. doi: 10.1371/journal.pone.0332367 (PMC12443314; doi:10.1371/journal.pone.0332367)
Supplement: S3 File — (PDF) [file pone.0332367.s003.pdf]

S3: Antioxidant assay of MgONPs.

Antioxidant (%)					
Ascorbic (µg/mL)	R1	R2	R3	Mean	SD
1000	98.3	97.5	97.2	97.66667	0.568624
500	92.36	92.7	91.3	92.12	0.730205
250	83.3	83.1	83.9	83.43333	0.416333
125	70.6	69.5	69.2	69.76667	0.737111
62.5	58.96	59.6	59.86	59.47333	0.463177
31.25	47.96	46.9	46.8	47.22	0.642806
15.63	39.6	39.2	39.1	39.3	0.264575
MgONPs (µg/mL)	R1	R2	R3	Mean	SD
1000	76.3	75.9	75.2	75.8	0.556776
500	64.39	63.5	63.1	63.66333	0.660328
250	52.36	51.9	52.7	52.32	0.401497
125	40.89	40.62	39.5	40.33667	0.737044
62.5	32.86	31.7	31.5	32.02	0.734302
31.25	23.6	23.1	22.5	23.06667	0.550757
15.63	10.9	10.5	10.2	10.53333	0.351188
