# Supplementary material for: Green synthesis, characterization, molecular simulation, and in vitro biomedical application of magnesium oxide nanoparticles
Source: PLoS One. 2025 Sep 17;20(9):e0332367. doi: 10.1371/journal.pone.0332367 (PMC12443314; doi:10.1371/journal.pone.0332367)
Supplement: S4 File — (PDF) [file pone.0332367.s004.pdf]

S4: Antiviral activity of MgONPs against HSV1 and HAV viruses.

Test	ug/ml	O.D			Mean O.D	±SE	Viability %	Toxicity %	Viral activity %	Ant iviral effect %
**Vero**	---	0.821	0.842	0.831	0.8313333	0.010504	100	0	---	---
**HSV1**	---	0.325	0.331	0.336	0.3306667	0.0055076	40.43	59.57	100	0
**MgO NPs**	62.5	0.59	0.576	0.582	0.5826667	0.0070238	70.036101	29.963899	50.300317	49.69968
	31.25	0.53	0.512	0.5102	0.5174	0.010949	61.395909	38.604091	64.804585	35.19541
	15.62	0.475	0.462	0.461	0.466	0.0078102	55.475331	44.524669	74.743443	25.25656
	7.81	0.41	0.402	0.403	0.405	0.0043589	48.495788	51.504212	86.459983	13.54002
	3.9	0.342	0.351	0.354	0.349	0.006245	42.599278	57.400722	96.358439	3.641561

Test	ug/ml	O.D			Mean O.D	±SE	Viability %	Toxicity %	Viral activity %	Ant iviral effect %
**Vero**	---	0.723	0.711	0.714	0.716	0.006245	100	0	---	---
**HAV**	---	0.322	0.334	0.329	0.3283333	0.0060277	45.81	54.19	100	0
**MgO NPs**	62.5	0.643	0.651	0.655	0.6496667	0.0061101	91.736695	8.2633053	15.248764	84.751236
	31.25	0.512	0.531	0.539	0.5273333	0.0138684	75.490196	24.509804	45.229385	54.770615
	15.62	0.432	0.442	0.439	0.4376667	0.0051316	61.484594	38.515406	71.074748	28.925252
	7.81	0.393	0.395	0.396	0.3946667	0.0015275	55.462185	44.537815	82.188255	17.811745
	3.9	0.345	0.346	0.351	0.3473333	0.0032146	49.159664	50.840336	93.818668	6.1813321
